# A new flea, *Ectinorus (Ectinorus) insignis* n. sp. (Siphonaptera, Rhopalopsyllidae, Parapsyllinae), with notes on the subgenus *Ectinorus* in Chile and comments on unciform sclerotization in the superfamily Malacopsylloidea

**DOI:** 10.1051/parasite/2013033

**Published:** 2013-10-15

**Authors:** Jean-Claude Beaucournu, Sorya Belaz, Sebastián Muñoz-Léal, Daniel González-Acuña

**Affiliations:** 1 Laboratoire de Parasitologie et Zoologie appliquée, Faculté de Médecine 2 avenue du Professeur Léon Bernard 35043 Rennes Cedex France; 2 Institut de Parasitologie de l’Ouest, Faculté de Médecine 2 avenue du Professeur Léon Bernard 35043 Rennes Cedex France; 3 Laboratoire de Parasitologie, Mycologie et Immunologie parasitaire, Centre Hospitalier Régional Universitaire 2 rue Henri Le Guilloux 32033 Rennes Cedex France; 4 Facultad de Ciencias Veterinarias, Universitad de Concepción Casilla 537 Chillán Chile

**Keywords:** Siphonaptera, Malacopsylloidea, Rhopalopsyllidae, Chile, New species

## Abstract

A list is provided for the species of *Ectinorus sensu stricto* from Chile. *Ectinorus* (*Ectinorus*) *insignis* n. sp. is described from Chile: this species is characterized by the male genitalia. In the subgenus *Ectinorus*, the authors report the presence in Chile of *E. pilosus* Beaucournu & Carmen Castro, 2002 described from Argentina and *E. simonsi* (Rothschild, 1904) described from Bolivia but also known from Peru. A female neallotype is designated for *E. ineptus* Johnson, 1957. “Unciform sclerotization” is noted and illustrated for the first time, in all Malacopsylloidea, and a list is given for all studied species.

## Abstract (fr)

La liste des *Ectinorus sensu stricto* connus du Chili est donnée et *Ectinorus* (*Ectinorus*) *insignis* n. sp. est décrit: l’espèce est caractérisée par la structure des genitalia mâles. Dans le sous-genre *Ectinorus*, les auteurs signalent la présence au Chili de *E. pilosus* Beaucournu & Carmen Castro, 2002, décrit d’Argentine et de *E. simonsi* (Rothschild, 1904) décrit de Bolivie, puis retrouvé au Pérou. Un néallotype femelle est désigné pour *E. ineptus* Johnson, 1957. Une “sclérification unciforme” est notée et illustrée pour la première fois chez tous les Malacopsylloidea, et une liste est donnée pour toutes les espèces étudiées.

## Introduction

The interest of the genus *Ectinorus* (and the related genera *Delostichus*, *Tetrapsyllus*, etc.), regardless of its morphological features, is that it is one of the bubonic plague vectors, transmitting *Yersinia pestis* in Chile-Andean subregion (e.g., Macchiavello, 1948) [28]. Admittedly, the importance of plague in the southern cone of South America is of lesser importance than in the Old World (Kazakhstan, Mongolia, China, Vietnam, Republic of Congo, Tanzania, Madagascar), but the potential for disease remains and a knowledge of endemic fleas is important.

## Genus *Ectinorus* Jordan, 1942

The genus *Ectinorus* (Rhopalopsyllidae: Parapsyllinae), endemic to the Chile-Andean subregion, includes related sigmodontine rodents fleas. Three subgenera are included in Smit’s review (1987) [36]: *Panallius* Jordan, 1942, *Ectinorus sensu stricto* and *Ichyonus* Smit, 1987. Until now 33 *Ectinorus sensu stricto* species were known (Hastriter & Sage, 2009) [16]. Fifteen species occur in Chile (those for which type localities are in Chile are marked with an asterisk) and include (listed alphabetically): E. chilensis* Lewis, 1976, E. cocyti* (Rothschild, 1904), E. curvatus* Beaucournu & Gallardo, 1991, E. gallardoi* Hastriter, 2001, *E. ineptus* Johnson, 1957 (in Beaucournu & Gallardo, 1991) [5], *E. ixanus* (Jordan, 1942) (in Beaucournu & Kelt, 1990) [9], E. lagidium* Beaucournu & Gonzàlez, 2005, *E. levipes* (Jordan & Rothschild, 1923) (in Beaucournu & Kelt, 1990), E. martini* Lewis, 1976, E. mimacydis* Beaucournu & Gallardo, 2004, E. mondacai* Hastriter, 2001, *E. nomisis* Smit, 1987 (in Beaucournu & Gallardo, 1989: description of female neallotype) [4], *E*. *setosicornis* Jordan, 1942 (in Beaucournu & Gallardo, 1991) [5], E. splendidus* Smit, 1968 and E. uncinatus* Beaucournu & Gallardo, 1991.

An additional three species are here reported from Chile for the first time: *Ectinorus pilosus* Beaucournu & Carmen Castro, 2002 [2] described from Argentina, *E. simonsi* (Rothschild, 1904) [32] known previously from Bolivia and Peru, and a new species, *Ectinorus insignis* Beaucournu & Gonzàlez-Acuña. In addition, a neallotype is designated for the female of *Ectinorus ineptus* Johnson, 1957 [19]. The total number of *Ectinorus sensu stricto* known from Chile is elevated to 18 species.

### *Ectinorus (Ectinorus) pilosus* Beaucournu & Carmen Castro, 2002

- *Ectinorus pilosus* Beaucournu & Carmen Castro, 2002: Description of holotype male and allotype female, Santa Maria (26° 40′ S–66° 02′ W) (Catamarca), Argentina, on *Ctenomys* sp. (later determined as *Ctenomys knighti* Thomas, 1919), collection date unknown.

For this species, one male and one female were found in Chile, Copiapó (Atacama), on *Ctenomys* sp., collection data unknown.

### *Ectinorus (Ectinorus) simonsi* (Rothschild, 1904)

- *Pulex simonsi* (Rothschild, 1904): two males and one female on *Neoctodon simonsi* (= *Octodontomys gliroides*) at Challapata (Avaroa, Bolivia), X/11/1901.
- *Rhopalopsyllus simonsi* (Rothschild): Jordan & Rothschild, 1908 [23]: redescription.
- *Dysmicus acheronis* Johnson, 1957 [19]: holotype male, Yuru (Arequipa, Peru) on *Galea musteloide*s, VIII/8/1939. Smit (1987): *acheronis* synonymized with *simonsi.*
- *Ectinorus* (*Ectinorus*) *simonsi* (Rothschild, 1904): drawings of male and female on *Lagidium viscacia* and a new locality is reported: Zudañez, Bolivia (in the Bolivian subregion, province of Jaime Zudañez), IV/19/1955 (Smit, 1987) [36].

One male found in Chile, Chusmiza (19° 40′ S–69° 10′ W), altitude 3430 m (Tarapaca), on *Octodontomys gliroides*, X/15/2011 (D. Gonzàlez-Acuña *rec*.). Identification is obvious for this species (apex of aedeagus, sternum VIII) but in this area on October 9, 1989, we found on the same host, at 2500 m altitude, three male intergrades between *simonsi* and *nomisis* Smit, 1987. These taxa are so close that it is likely that *E. nomisis* is a subspecies of *E. simonsi.*

### *Ectinorus (Ectinorus) ineptus* Johnson, 1957

- *Ectinorus ineptus* Johnson, 1957: description of male holotype (plates 67–69) at Picotani (Puno, Peru), on *Phyllotis* (*Auliscomys*) *pictus*, IX/14/1941.
- *Ectinorus* (*Ectinorus*) *ineptus* Johnson 1957: Smit (1987) illustrated the genitalia of the male holotype (their Figures 189 and 190), from *Auliscomys pictus*.
- *Ectinorus* (*Ectinorus*) *ineptus* Johnson, 1957, Beaucournu & Gallardo, 1991 [5]: Parinacota (18° 12′ S–69° 16′ W), altitude 4000 m, Chile, on *Phyllotis darwini*, IX/30/1989. One male and one female: Short description of the male and the female (Figures 6, 9, 12–14), female explicitly “not designated as neallotype because two other very close taxa have been collected in the same region syntopically with this species (e.g., *E. curvatus* n. sp.)”.
- *Ectinorus* (*Ectinorus*) *ineptus* Johnson, 1957: Lake Chungara (18° 15′ S–69° 10′ W), altitude 4574 m, Chile (Parinacota) on *Auliscomys boliviensis* (Waterhouse, 1846), one male and one female XI/21/2011; Enquelga, altitude 3700 m, Chile (Tarapaca) on *Eligmodontia puerulus* (Philipi, 1896), one female X/17/2011 (D. Gonzàlez-Acuña *rec*.) (The determinations of all Chilean hosts are after Wilson & Reeder, 2005) [37].

So we have two pairs, each in syntopy on the same individual host and a sole female: therefore, the identity of the female drawn in 1991 is confirmed. Currently males, in Chile, have been collected from two locations close to each other.

We select the 1991 female, as neallotype, because the others have misplaced spermatheca, but are otherwise identical. So the neallotype is from Parinacota (18° 12′ S–69° 16′ W), altitude 4000 m, Chile (Parinacota) on *Phyllotis darwini*, IX/30/1989.

#### Description of females

Material examined: two males and three females, as indicated above.

*Material deposited*: Neallotype in the collection of first author (Collection JCB currently in Faculty of Medicine, Rennes), for ultimate deposition in Muséum National d’Histoire Naturelle, Paris, France (MNHN).

*Head capsule*: With well-developed tubercle, without micro-seta visible in the vallum. Genal margin slightly convex; maxillary palpus with segments I, II and IV of same length; segment III shorter; labial palpus with seven segments, not reaching apex of coxa; pre-antennal seta very small; six marginal setae and two small, thicker setae in posterior point of the gena; two occipital rows, respectively, of one and five setae.

*Thorax*: Prothorax with anterior row of five small setae and posterior row of seven with intercalaries. Mesothorax: 14 small setae and principal row of 6 longer setae; 10 pseudosetae. Metathorax: four anterior setae and posterior row of seven longer setae, most dorsal seta curved. Metepimeron with four long setae; spiracle pointed, or shaped as a “nightcap”. Femoral guard setae long; external half the length of internal. Tibia with eight notches on dorsal margin. Notches II, V and VIII with, at least, one long seta. Longest seta of notch VIII extends to apex of first tarsomere. Longest seta of this tarsomere extends to apex of tarsomere II; longest seta of tarsomere II extends to half of the length of distitarsomere.

**Figures 1–3.**
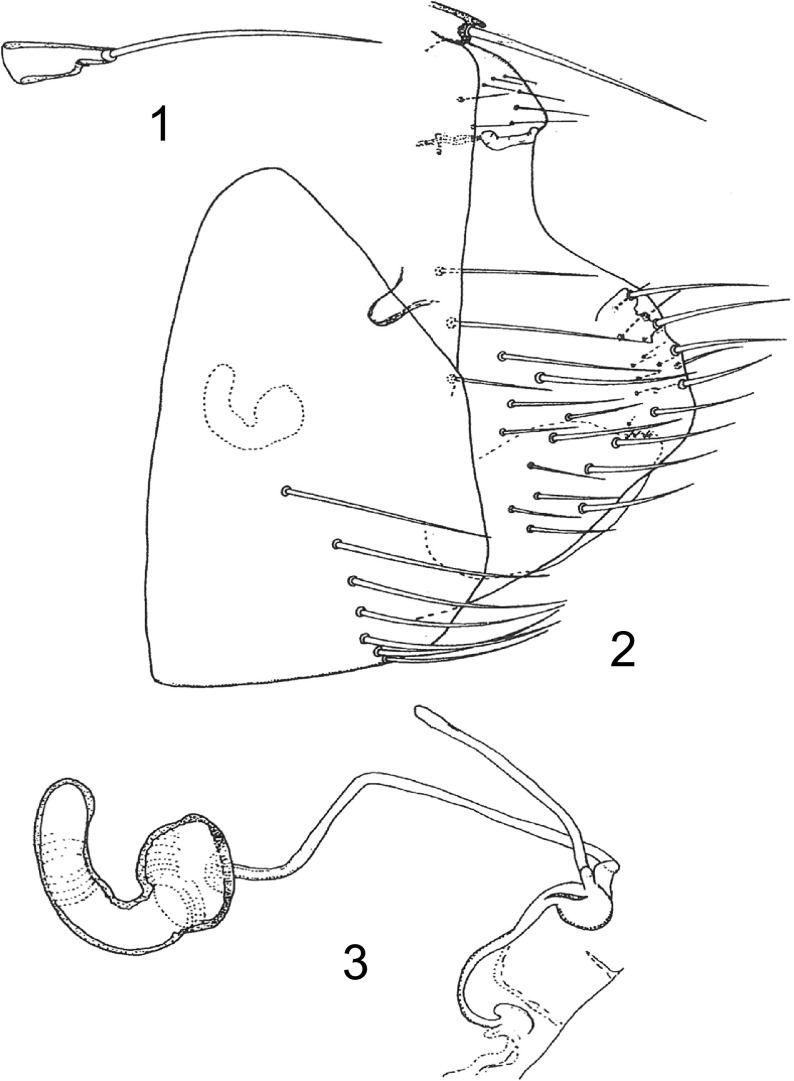
*Ectinorus (Ectinorus) ineptus* (Johnson, 1957) neallotype. 1: anal stylet; 2: terminal segment and unciform sclerotization; 3: spermatheca and *ducti*.

*Abdominal segments*: Tergum I with eight spinelets, four on each side. Tergum II with row of seven long setae, most ventral seta at level of spiracle. Same setation on terga III to VII, but most ventral seta below spiracles; spiracles relatively large and rounded. Tergum VIII (Figure 2) with small area of seven or eight small setae anterior to spiracle; spiracle marginal and vermiform. Unciform sclerotization present (see below). Anal stylet (Figure 1) long, conical, with one small seta below insertion of long apical seta. Below ventral anal segment four long marginal setae and 16–18 lateral setae of various lengths and seven to eight thin, small mesal setae. Sternum II with 1 barely discernible striarium and laterally 17 thin lateral setae and 2 small marginal setae. Sternum III with lateral row of six setae and three to four dispersed setae. Sterna IV and V with main row of four or five setae and one isolated seta. Sternum VI with simple row of five setae. Sternum VII (Figure 2) with row of six setae inserted on half of the segment. Margin gently convex. Sternum VIII (Figure 2) large with four to five apical micro-setae.

*Spermatheca*: Bulga angular (Figure 3), slightly wider than long; hilla slightly curved, twice as long as bulga; *ductus obturatus* and *ductus spermathecae* of same width, the *d. s*. twice as long as the *d. o*. Typically, for this group, *ductus bursa* appears as a curved “*ε*”.

Dimensions (slide-mounted insects in personal collection): males 2.1–2.8 mm (holotype 2.7), females 2.5–2.8 mm.

*Specificity*: *Auliscomys* Osgood, 1915 (Rodentia – Cricetidae – Sigmodontinae) must be the elective type host-genus; it is taxonomically very close to *Phyllotis* Waterhouse, 1837.

### *Ectinorus* (*Ectinorus*) *insignis* Beaucournu & González-Acuña n. sp.

urn:lsid:zoobank.org:act:31EF7F63-C723-4961-8628-7548325BEAB6

*Authorship*: Note that the authors of the new taxon are different from the authors of this paper; Article 50.1 and Recommendation 50A of International Code of Zoological Nomenclature.

*Material examined*: Holotype male on *Eligmodontia puerulus* (Philippi, 1896) (Rodentia – Cricetidae – Sigmodontinae), Toconao (23° 07′ S–68° 00′ O), altitude 2469 m, (El Loa), Chile, X/11/2011. Allotype female in syntopy with the holotype (D. González-Acuña *rec*.).

*Material deposited*: Types in the collection of first author (Collection JCB, currently in Faculty of Medicine, Rennes), for ultimate deposition in Muséum National d’Histoire Naturelle, Paris (MNHN).

*Etymology*: from the Latin word *insignis* derived from *in signum* “showing a particular character”, this in reference to the male genitalia.

#### Description

*Ectinorus insignis* n. sp. has a well-developed *processus basimeris ventralis* and belongs to the “*hapalus*-group” (Smit, 1987) [36]. From a dichotomous view, this character is not chosen by this author who uses the presence or absence of spinelets on tergum I.

*Head capsule*: frontal tubercle not very prominently situated a short distance from the labial angle. Maxillary and labial palps extend slightly beyond the apex of trochanter. Segments I and III of maxillary palpus are the same length but slightly shorter than II or IV. Labial palpus with eight segments, including basal segment. Pre-antennal seta of male well developed, but very thin and small in female. Three pre-ocular setae, the ventral one longer as typical. Eye pigmented, indented and with sclerotized “bridge” near the *gena.* One post-ocular seta, three marginal setae and one very small spiniform seta on angle between *gena* and antennal fossa. three occipital rows with one, one and four setae in the male but only two rows in the female of one and four setae.

*Thorax*: prothorax with one marginal row of six well-developed setae and one anterior row of three. Male with single pseudoseta, female with two. Mesothorax with one marginal row of four long setae and anteriorly three to four small ones; one row of pseudosetae, 13 in the male, 12 in the female. Metathorax with one marginal row of four well-developed setae. Metepimeron with four setae and the spiracle shaped like a “nightcap”. Femur I: femoral guard setae very unequal in length (internal seta length and thickness twice that of the external one). Femur III: internal femoral-tibia guard seta four times as long as the external one but only slightly thicker. Tibia III with six dorsal notches bearing setae, the second, fourth and sixth with at least one long slender seta. First tarsomere III with a long apical seta as long as this segment. Second tarsomere III with a tuft of three long setae, the longest extending beyond the apex of the distitarsomere in the male and half that length in the female.

*Abdomen (unmodified segments)*: male tergum I without spinelet while the female has one or two small spinelets. Terga II–VII, one principal row of six setae, the most ventral at level of spiracle in the male but below the spiracle in the female. Spiracles practically round in the male whereas shaped like a «nightcap» in the female. One ante-sensilial seta; marginal in the female, as typical. Sensilium typical with 17–18 pits. Sternum II with two thin marginal setae; three thin lateral setae (male) or 16–18 (female). Striarium absent. Sterna III and IV with row of three thin setae (male) or two to three (female). Sterna V–VII with one row of two thin setae (male) or respectively three, five and six long, thin and gently curved setae (female).

**Figures 4–10.**
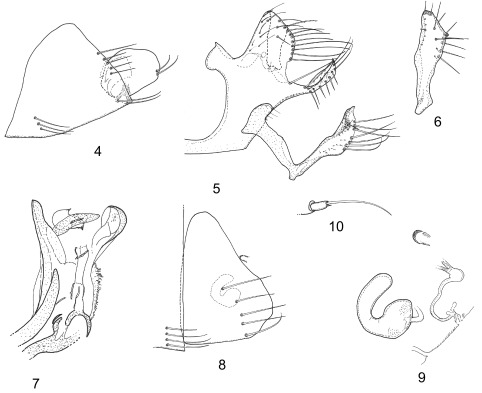
*Ectinorus (Ectinorus) insignis* Beaucournu & González-Acuña n. sp. Holotype, 4: sternum VIII; 5: segment IX; 6: p*rocessus telomeris*; 7: phallosome apex – Allotype; 8: sternum VII; 9: unciform sclerotization, spermatheca and *ducti*.; 10: anal stylet.

*Abdomen (male genital segments)*: Sternum VIII (Figure 4) with inferior-distal lobe as in numerous species of *Ectinorus*. Lobe, circular with two small curved setae on its posterior margin, linked to the segment by a well-sclerotized connection (in lateral aspect). Segment IX (Figure 5) with very long *processus basimeris ventralis* appears longer due to lack of setae on posterior-margin of basimere.

*Processus basimeris* or Basimere: anterior and posterior edges subequal. About 10 thin setae on anterior and external borders. Margin of posterior border with one row of nine setae (slightly thicker than the drawing). Below this row, border is hyaline and naked. Inferior margin with thin setae. Ventral margin of manubrium forms a straight line visible but not as marked as in *E. ineptus*. *Processus basimeris ventralis* narrow, relatively long, with an acuminate apex; apex is anteriorly folded up as in a penknife. External part with three long and six shorter setae on its lateral margin. Internal part with only small setae. Dorsal margin of p.b.v., with two long setae; most anterior seta at base of this process

*Processus telomeris* (Figure 6) or Telomere, entirely masked by basimere, not extending beyond basimere as in *simonsi* and *nomisis*. Telomere longer than broad with triangular apex posterior end forming a short forward process.

*Sternum IX* (Figure 5): Proximal arm broad basally, narrower ventrally; distal arm narrow, then broader with a pointed apex. One short median seta and seven long curved setae on ventral margin. Tuft of six to seven small thin setae on internal surface.

*Phallosome* (Figure 7): classical structure for this genus, and subgenus, but apico-ventral lobe particularly broad.

*Abdomen (female genital segments)*: Tergum VIII with four to five small setae above spiracle, spiracle elongated and vermiform; laterally about 12 small setae. Unciform sclerotization visible above *spermatheca*, on tergum VIII, shaped like a “horseshoe” and slightly pigmented (Figures 8 and 9).

*Sternum VII* (Figure 8): posterior margin convex on ventral half with five long setae remote margin; no other seta. Anal stylet (Figure 10) cylindrical with one apical seta long and gently curved, and basally one very small seta. Sternum VIII longer than wide (and very different from *E. ineptus* in this regard).

*Spermatheca* (Figure 9): bulga somewhat quadrangular; *area cribriformis* in the middle of proximal end; hilla broad and subrectangular. *Ductus bursae* with shape of a cursive *ε*, characteristic of this species-group, for example in *ineptus, convexus, hirsutus*, etc.

*Dimensions* (slide-mounted specimens): holotype male 1.9 mm; allotype female 2.7 mm.

#### Diagnosis

Among the species of *Ectinorus sensu stricto*, *E. ineptus* Johnson, 1957 [19], *E. hirsutus* Hastriter, 2009 [15] and *E. splendidus* Smit, 1968 [34] show some similarities with *E. insignis* n. sp. These species belong to *Ectinorus* having marginal spinelets on tergum I, they also belong to the *hapalus*-group having *processus basimeris ventralis*. This group shares common characters with the former genus *Dysmicus* and the genus *Ectinorus sensu stricto* (Smit, 1987) [36].

A non-genital difference is noted for the first time. *Ectinorus* are split into two groups by Smit [36] with respect to whether or not they have spinelets on the margin of tergum I. As we have stated, *E. insignis* n. sp. belongs to the *hapalus-*group, having *processus basimeris ventralis*, but unlike *ineptus* and *hirsutus* the male has no spinelet on tergum I and the female has only one or two instead of the more typical, four or six. Indeed, *Dysmicus* Jordan, 1942 [21] is characterized by no spinelet on tergum I, seems to mirror *Ectinorus,* in this respect.

*Segment IX*: Basimere has the same general shape as in the taxa previously mentioned. However in *insignis* it is less compact; the posterior margin and chaetotaxy are also different. *E.* s*plendidus*, *hirsutus* and *insignis* have almost the same *processus basimeris ventralis* (pbv), but in *E. insignis* it is straight and not bent at right angles as in *E. hirsutus*. The pbv is short, massive and inserted at the base of basimere in *E. ineptus.* Among *E. Ectinorus*, only *E. splendidus* and *E. insignis* share-related shapes of the pbv. Sternum IX is close to that of *E. ineptus* by its pointed apex. The lobe of sternum VIII is not present in *E. splendidus*. This lobe is almost circular in *E. insignis* and in *E. ineptus*. However it bears only two setae in *E. insignis* while there are eight or nine in *E. ineptus* (six or eight in our specimens); *E. hirsutus* also has a circular ventrally indented lobe. For *E. insignis*, sternum VII only has five setae adjacent from the margin, whereas there are 10–11 setae (11 and 12 in our specimens) close to the margin in *E. ineptus* and *E. hirsutus*. The sternum of *E. splendidus* is very different.

Females are very similar to each other (the female of *E. splendidus* is currently unknown) and always have unciform sclerotization.

*Host specificity*: At this time, *Ectinorus insignis* n. sp. is only known from *Eligmodontia puerulus*, a sigmodontine rodent, which is also a secondary host of *Ectinorus ineptus*, as cited above.

## Unciform sclerotization

**Figures 11–25:**
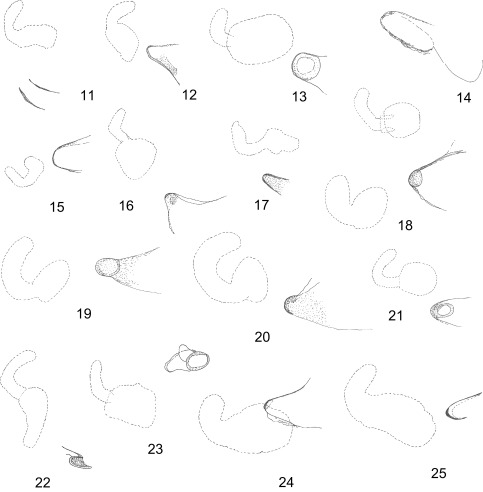
Unciforms sclerotizations in various species (the drawing of the outline of the spermathaeca is also included for scale and site of sclerotization) – Ctenophthalmidae, Ctenophthalminae: 11: *Ctenophthalmus* (*Ethioctenophthalmus*) *eximius* Jordan & Rothschild, 1913, Tanzania; 12: *C.* (*Euctenophthalmus*) *savii calabricus* Beaucournu, Valle & Launay, 1981, Italia – Malacopsyllidae: 13: *Malacopsylla grossiventris* (Weyenbergh, 1879), Argentina; 14: *Phthiropsylla agenoris* (Rothschild, 1904), Argentina – Rhopalopsyllidae, Parapsyllinae: 15: *Delostichus coxalis* (Rothschild, 1909), Chile; 16: *Tetrapsyllus* (*Tetrapsyllus*) *maulinus* Beaucournu & Gallardo, 1978, Chile; 17: *Tetrapsyllus* (*Heteropsyllus*) *satyrus* Beaucournu & Torres-Mura, 1986, Chile; 18: *Listronius plesiomorphus* Beaucournu & Gallardo, 1991, Chile; 19: *Parapsyllus senellarti* Beaucournu & Rodhain, 1990, Amsterdam Island; 20: *Parapsyllus nestoris antichthones* Smit, 1979, Antipodes Island – Rhopalopsyllinae: 21: *Rhopalopsyllus lugubris lugubris* Jordan & Rothschild, 1908, French Guyana; 22: *Tiamastus helicis* Beaucournu & Carmen Castro, 2003, Argentina; 23: *Polygenis* (*Gephyropsylla*) *klagesi klagesi* (Rothschild, 1904), French Guyana; 24: *Polygenis* (*Polygenis*) *rimatus* (Jordan, 1932), Brasil; 25: *Polygenis* (*Neopolygenis*) *pradoi* (Wagner, 1937), Colombia.

These are “sclerotised fold (or folds) on the anterior portion of tergum VIII of the female in some fleas, usually situated under sternum VII and near its margin” (Rothschild & Traub, 1971) [31], studied also by Smit (1970) [35] and Peus (1976) [30]. They were first drawn by Jordan (1936) [20] with the description of *Ctenophthalmus singularis* (presently *C.* (*Ethioctenophthalmus*) *singularis*), then quoted by Smit (1963) [33] in a “Species-group in *Ctenophthalmus*” and quoted again by Hopkins & Rothschild (1966) [17] in volume IV of “the Catalogue of the Rothschild collection of Fleas (Hystrichopsyllidae: Ctenophthalminae)” (now Ctenophthalmidae) and finally thoroughly described by Peus (op. cit.) always in various *Ctenophthalmus* Kolenati, 1856: as in the subgenera *Ethioctenophthalmus* Hopkins & Rothschild, 1966 (Figure 11), *Euctenophthalmus* Wagner, 1940 (Figure 12) and *Metactenophthalmus* Peus, 1976. Among *Ctenophthalmus*, on one side, the “sclerotisations”, as spelled by Rothschild & Traub (1971) [31], are rarely described in *Ethioctenophthalmus* a highly polymorphic subgenus which is undoubtedly polyphyletic, on the other hand, they are obvious in *C. (E.) leptodactylus* Hubbard, 1963, more discreet in *C. (E.) smithersi* De Meillon, 1952. However, they are always seen in *Euctenophthalmus* and their relatives in *Metactenophthalmus*.

As Peus [30] showed, these sclerotizations are complex. Depending on the species or genus, the structures will be more or less visible. So the large differences noted in forms are not surprising, for example between Ctenophthalmidae and Malacopsylloidea. This structure is also present in a number of other Families: for instance, slerotization is visible in two widely distributed species, *Leptopsylla segnis* (Schönherr, 1811) and *Nosopsyllus fasciatus* (Bosc d’Antic, 1800), Ceratophyllidae (respectively Leptopsyllinae and Ceratophyllinae) and shows similarities with those of Rhopalopsyllinae (Beaucournu, unpublished).

In the Superfamily Malacopsylloidea, even though they are consistent in a given species, unciform sclerotizations are random with respect to genus or other taxa. Their shape varies from one species or genus to another. In our opinion, the interest is not only in their shape but also their presence or absence. In the genus *Ectinorus*, Smit (1987) [36] draws an unciform sclerotization, without comment, for some species such as *E. levipes* (Jordan & Rothschild, 1923), *E*. *viscachae* (Wagner, 1937) (e.g., *Ectinorus*) and *E. onychius* (Jordan & Rothschild, 1923) (s. g. *Ichyonus*). But, this sclerotization is also visible in *Ectinorus barrerai* Jordan, 1939, *E chilensis* Lewis, 1976, *E. cocyti* (Rothschild, 1904), *E. ixanus* (Jordan, 1942), *E. martini* Lewis, 1976 and *E. setosicornis* Jordan, 1942. However, Smit [36] seems to have overlooked this structure when he studied these taxa. Thereafter, some species with the sclerotization were described after the publication of Smit’s “Monograph”. In *E. uspallatae* Beaucournu & Gallardo, 2005 [6], for example, reported it as a lacuna, a name proposed by Peus [30] for some of these structures. Conversely, it is lacking in *E.* (*Panallius*) *galeanus* (Jordan, 1939).

Unciform sclerotization exists in other Rhopalopsyllidae. Smit [36] drew it, for example, in various *Parapsyllus* and in Malacopsyllidae. This family is composed of two monotypic genera, *Malacopsylla* Weyenberg, 1879 and *Phthiropsylla* Wagner, 1939. Rhopalopsyllidae are relatives of Malacopsyllidae and form with them the Superfamily Malacopsylloidea.

Smit (1987) [35] and various authors (including Johnson, 1957 [19], Linardi & Guimarães, 1993 [26], 2000 [27]) have drawn them in Rhopalopsyllidae. But the discussion must extend beyond these drawings, because the authors did not seem interested in the structure. However, Linardi & Guimarães (2000) [27] point out “*mancha escura*” (dark spot) or “*mancha clara escamiforme*” (scaly bright spot) in several Rhopalopsyllinae. Those are various unciform sclerotizations.

Combining these data and ours, we report unciform sclerotization for Malacopsylloidea in Table 1.

**Table 1.** Presence or absence of unciform sclerotization in the superfamily Malacopsylloidea.

Family	Subfamily	Genus	Subgenus	Species/subspecies	Presence or absence of unciform sclerotization
Malacopsyllidae		*Malacopsylla* Weyenberg, 1881		*grossiventris* 1 (Weyenberg, 1879) (Figure 13)	Presence Visible in Smit’s drawings (1987) [35]
		*Phthiropsylla* Wagner, 1939		*agenoris* 1 (Rothschild, 1904) (Figure 14)	
Rhopalopsyllidae	Parapsyllinae	*Delostichus* Jordan, 1942		*ojedoi* Beaucournu & Gallardo, 2005 *smiti* Jameson & Fulk, 1977 [18] *talis* (Jordan, 1936) *xenurus* (Rothschild, 1914)	Females not studied or unknown Presence or absence undeterminable
				*coxalis* (Rothschild, 1909) (Figure 15) *degus* Beaucournu, Moreno & González-Acuña, 2011 [10] *incisus* Beaucournu & Torres-Mura, 1987 [12] *octomyos* Jordan, 1942 [22] *phyllotis* Johnson, 1957 [19]	Presence Sometimes difficult to see in some *coxalis* and *incisus*, but these specimens may have been overcleared
		*Tetrapsyllus* Jordan, 1931	*Phylliver* Smit, 1987	*bleptus* (Jordan & Rothschild, 1923) Females provisionally assigned to *elutus* Johnson, 1957	Presence Males not studied
			*Tetrapsyllus* s. sto	*comis* Jordan, 1931 *contortrix* Jameson & Fulk, 1977 *tristis* Johnson, 1957 [19]	Females not studied Presence or absence undeterminable
				*amplus* (Jordan & Rothschild, 1923) *corfidii* (Rothschild, 1904) *maulinus* Beaucournu & Gallardo, 1978 (Figure 16) *rhombus* Smit, 1955 *tantillus* (Jordan & Rothschild, 1923)	Presence Sclerotization in *T. rhombus* is visible in Figure 117 of Smit (1987) [36]
			*Heteropsyllus* Beaucournu, 2002 [1]	*incisus* 1 Beaucournu & Torres-Mura, 1986 (Figure 17) [11]	Presence
Rhopalopsyllidae	Parapsyllinae	*Ectinorus* Jordan, 1942	*Panallius* Jordan, 1942	*galeanus* 1 (Jordan, 1939)	Absence
			*Ectinorus* s. sto.	*barrerai* Jordan, 1939 *chilensis* Lewis, 1976 [25] *cocyti* (Rothschild, 1904) *curvatus* Beaucournu & Gallardo, 1991 *gallardoi* 2 Hastriter, 2001 [14] *hirsutus* Hastriter, 2009 *ineptus* Johnson, 1957 (Figure 1) *insignis* n. sp. (Figure 10) *ixanus* (Jordan, 1942) *levipes* (Jordan & Rothschild, 1923) *martini* Lewis, 1976 [25] *nomisis* Smit, 1987 *pilosus* Beaucournu & Carmen Castro, 2002 *setosicornis* Jordan, 1942 (*E. simonsi* Rothschild, 1904, not examined but included with *nomisis* (see above)) *splendidus* Smit, 1968 *uspallatae* Beaucournu & Gallardo, 2002 *viscachae* (Wagner, 1931)	Presence Female of lagidium Beaucournu & Gallardo, 2005 [8] is unknown Female of mimacydis Beaucournu & Gallardo, 2004 [7] is unknown
			*Ichyonus* Smit, 1987	*onychius onychius* (Jordan & Rothschild, 1923)	Presence Visible in Smit’s drawings (1987) [35]
		*Eritranis* Jordan, 1942		*andricus* 1 (Jordan, 1939)	Presence
		*Listronius* Jordan, 1942		*fortis* (Jordan & Rothschild, 1923) *plesiomorphus* Beaucournu & Gallardo, 1991 (Figure 18) *robertsianus* 2 (Jordan, 1938) *ulus* (Jordan & Rothschild, 1923)	Presence
		*Parapsyllus* Enderlein, 1903		cardinis* Dunnet, 1961 *dacunhai* De Meillon, 1952 *heardi* De Meillon, 1952 *humboldti* Jordan, 1942 jacksoni* Smit, 1965 longicornis* (Enderlein, 1901) lynnae lynnae* Smit, 1965 lynnae alynnae* Smit, 1979 magellanicus magellanicus* Jordan, 1938 nestoris nestoris* Smit, 1965 *nestoris antichtones* Smit, 1979 (Figure 20) (*cardinis* group) *senellarti* Beaucournu & Rodhain, 1990 (Figure 19) *struthophilus* Smit, 1979 valedictus* Smit, 1979 (*longicornis* group)	Presence *indicate Smit’s drawings (1965, 1979, 1987) with unciform sclerotization For *P*. *longicornis*, note that Christchurch’s females (from Amsterdam Island) clearly show unciform sclerotization while *P. longicornis* from Isla Magdalena (Southern Chile) do not or barely show it.
	Rhopalopsyllinae	*Rhopalopsyllus* Baker, 1905		*australis australis* (Rothschild, 1904) *australis tamoyus* Jordan & Rothschild, 1923* [24] *lugubris lugubris* Jordan & Rothschild, 1908 (Figure 21)* *lutzi lutzi* (Baker, 1904)* *saevus* Jordan & Rothschild, 1923 [24]	Presence *indicate a “mancha escura” reported by Linardi & Guimarães (2000) [27]
				*garbei* (Guimarães, 1940)	No or little sclerotization
		*Tiamastus*, Jordan, 1939		*callens* (Jordan & Rothschild, 1923) [24] *cavicola* (Weyenbergh, 1881) *deflatus* Smit, 1987* *gallardoi* Beaucournu & Kelt, 1990 [9] *helicis* Beaucournu & Carmen Castro, 2003 (3) (Figure 22) *palpalis* (Rothschild, 1911)* *plesius* Jordan, 1942* *subtilis* (Jordan & Rothschild, 1923) [24] *tortuosus* Beaucournu & Carmen Castro, 2003 [3]	Presence *indicate Smit’s drawings (1987) [36] with unciform sclerotization
		*Scolopsyllus* Méndez, 1968		*colombianus* ^1, 2^ Méndez, 1968 [29]	Neither the original description nor Smit show sclerotisation
		*Hechtiella* Barrera, 1952^2^		*nitidus* 2 Johnson, 1957	Presence on Figure 190 in Linardi & Guimarães (2000) [27]
				*lakoi* 2 Guimarães, 1948 *lopesi* 2 Guimarães & Linardi, 1993	
		*Neotropsylla* Linardi & Guimarães, 1993 [26]		*guimaraesi* ^1, 2^ Linardi, 1978	
		*Polygenis* Jordan, 1939	*Gephyropsylla* Barrera, 1952	*klagesi klagesi* (Rothschild, 1904) (Figure 23) *klagesi rangeli* 2 Smit, 1987 *klagesi samuelis* (Jordan & Rothschild, 1923) [24]	
			*Ayeshaepsylla* Smit, 1987	*thurmanni* 2 Johnson, 1957	Presence in all taxa studiedSmit (*op. cit*.) [36] shows no sclerotization for any of these species or subspecies
			*Neopolygenis* Linardi & Guimarães, 1993 [26]	*atopus* (Jordan & Rothschild, 1922) *dentei* 2 Guimarães, 1947 *frustratus* Johnson, 1957 *massoiai* Del Ponte, 1967 *pradoi* (Wagner, 1937) (Figure 25) *pygaerus* 2 (Wagner, 1937)	Linardi & Guimarães (*op. cit.*) [27] note a “*mancha escura*” for: *P. klagesi klagesi* *P. adelus* *P. atopus* And a “*mancha clara escamiforme*” for *P. tripus*These are unciform sclerotizations recognizable on photographs (Figure 171, 211, 302 and 330 in Linardi & Guimarães, 2000) [27]
Rhopalopsyllidae	Rhopalopsyllinae	*Polygenis* Jordan, 1939	*Polygenis* Jordan, 1939	*acodontis* 2 (Jordan & Rothschild, 1923) [24] *adelus* 2 (Jordan & Rothschild, 1923) [24] *adocetus* 2 Traub, 1950 *axius axius* (Jordan & Rothschild, 1923) [24] *axius pessoai* 2 Guimarães, 1956 *axius proximus* 2 Guimarães, 1948 *bolhsi bolhsi* (Wagner, 1901) *bolhsi jordani* (Da Costa Lima, 1937) *brachinus* Jordan, 1950 *caucensis* 2 Méndez, 1977 *delpontei* Méndez, 1977 *dendrobius* 2 (Wagner, 1939) *dunni* (Jordan & Rothschild, 1922) *floridanus* 2 Johnson & Layne, 1961 *gwyni* (Fox, 1914) *guimaraesi* 2 Linardi, 1978 *hopkinsi* 2 Méndez, 1977 *impavidus* 2 Johnson, 1957 *litargus* 2 (Jordan & Rothschild, 1923) [24] *litus* 2 (Jordan & Rothschild, 1908) *martinezbaezi* 2 Vargas, 1951 *occidentalis occidentalis* 2 (Cuhna, 1914) [13] *occidentalis steganus* 2 (Jordan & Rothschild, 1923) [24] *odiosus* 2 Smit, 1958 *platensis* (Jordan & Rothschild, 1908) *peronis* 2 (Jordan & Rothschild, 1923) *puelche* 2 (Del Ponte, 1963) *rimatus* (Jordan, 1932) (Figure 24) *roberti beebei* (Fox, 1947) *roberti roberti* 2 (Rothschild, 1905) *roberti tripopsis* 2 Guimarães, 1948 *rozeboomi* 2 Vargas, 1952 *trapidoi mendezi* 2 Smit, 1987 *trapidoi trapidoi* 2 Méndez, 1977 *tripus* 2 (Jordan, 1933) *vazquezi* 2 Vargas, 1951	Presence in all taxa studied Smit (*op. cit*.)[35] shows no sclerotization for any of these species or subspeciesLinardi & Guimarães [27] note a “*mancha escura*” for: *P. klagesi klagesi* *P. adelus* *P. atopus* And a “*mancha clara escamiforme*” for *P. tripus* These are unciform sclerotizations recognizable on photographs (Figure 171, 211, 302 and 330 in Linardi & Guimarães, 2000) [27]

1 Monotypic genus.

2 Taxa not studied.

In conclusion, there is unciform sclerotization for the two genera of Malacopsyllidae. In Rhopalopsyllidae – Parapsyllinae, it is present for every species of *Delostichus*, *Tetrapsyllus*, *Ectinorus*, *Eritranis* and *Listronius* studied. However, curiously, the enigmatic *Ectinorus* (*Panallius*) *galeanus* does not have it. Finally, it seems to be present in all species and subspecies of the subfamily Rhopalopsyllinae in the Rhopalopsyllidae. This structure varies in size and shape depending on the species but its presence can help species classification. We did not aim to search for the slerotization in all Siphonaptera, but hopefully by this note, we will draw attention to this structure by other researchers. Indeed the structure appears to have taxonomic value in the classification of particular subgenera within the genus *Ctenophthalmus*. Females, for example in Rhopalopsyllidae, are difficult to identify. Therefore, the presence or absence of sclerotization must be well figured or at least reported to aid in identification.
